# Vaccination directed against the human endogenous retrovirus-K (HERV-K) gag protein slows HERV-K gag expressing cell growth in a murine model system

**DOI:** 10.1186/1743-422X-11-58

**Published:** 2014-03-26

**Authors:** Benjamin Kraus, Katrin Fischer, Katja Sliva, Barbara S Schnierle

**Affiliations:** 1 Paul-Ehrlich-Institut, Department of Virology, Paul-Ehrlich-Straße 51-59, Langen 63225, Germany

**Keywords:** Human endogenous retrovirus, Gag, MVA

## Abstract

**Background:**

Human endogenous retroviruses (HERVs) are remnants of ancestral infections and chromosomally integrated in all cells of an individual, are transmitted only vertically and are defective in viral replication. However enhanced expression of HERV-K accompanied by the emergence of anti-HERV-K-directed immune responses has been observed inter-alia in HIV-infected individuals and tumor patients. Therefore HERV-K might serve as a tumor-specific antigen or even as a constant target for the development of an HIV vaccine.

**Results:**

To verify our hypothesis, we tested the immunogenicity of HERV-K Gag by using a recombinant vaccinia virus (MVA-HK_con_) expressing the HERV-K Gag protein and established an animal model to test its vaccination efficacy. Murine renal carcinoma cells (Renca) were genetically altered to express E. coli beta-galactosidase (RLZ cells) and the HERV-K Gag protein (RLZ-HKGag cells). Subcutaneous application of RLZ-HKGag cells into syngenic BALB/c mice resulted in the formation of local tumors in MVA vaccinated mice. MVA-HK_con_ vaccination reduced the tumor growth. Furthermore, intravenous injection of RLZ-HKGag cells led to the formation of pulmonary metastases. Vaccination of tumor-bearing mice with MVA-HK_con_ drastically reduced the number of pulmonary RLZ-HKGag tumor nodules compared to vaccination with wild-type MVA.

**Conclusion:**

The data demonstrate that HERV-K Gag is a useful target for vaccine development and might offer new treatment opportunities for cancer patients.

## Background

HERVs are relics of ancient viral infections events into the germ line and are since then transmitted vertically. These retrovirus genomes are chromosomally integrated in all cells of an individual and their sequences comprise about 8% of the human genome. HERVs are historically classified by the single letter amino acid code for the tRNA specific of the primer binding site used to initiate reverse transcription. Integrated endogenous retrovirus genomes commonly contain mutations, deletions or are reduced to a single LTR element, and so have mainly lost the ability to be transferred. In contrast, HERV-K is the only known endogenous retrovirus encoding all structural and enzymatic proteins (Gag, Prt, Pol), as well as an envelope protein (Env) and an accessory protein Rec with functional similarity to the HIV Rev protein. Despite this, HERV-K is not infectious and its gene expression is generally repressed. However, reactivation of HERV-K proviruses coding for all viral proteins occurs under certain circumstances and is well established for human teratocarcinomas [1], melanomas [2] and ovarian cancer [3,4]. The overexpression of Gag has been seen in the peripheral blood cells of leukemia patients [5] and also in prostate cancer and ovarian cancer but not in healthy donors [6,7].

Tumor-associated antigens (TAAs), proteins expressed mainly or exclusively by tumor cells, can be used for therapeutic vaccinations, in particular for the treatment of minimal residual disease. The induction of immunological memory may even prevent disease relapse. Recombinant poxviruses are frequently used as vaccine vectors, because they activate robust cellular MHC class I- and II-restricted CD8+ and CD4+ T cell responses against recombinant antigens [8].

Here we tested a recombinant vaccinia virus expressing the HERV-K Gag gene for its efficacy in elucidating a cytotoxic immune response, which might be beneficial as a novel cancer vaccine.

## Results

### Establishment of an animal model and the experimental vaccine

**Figure 1:**
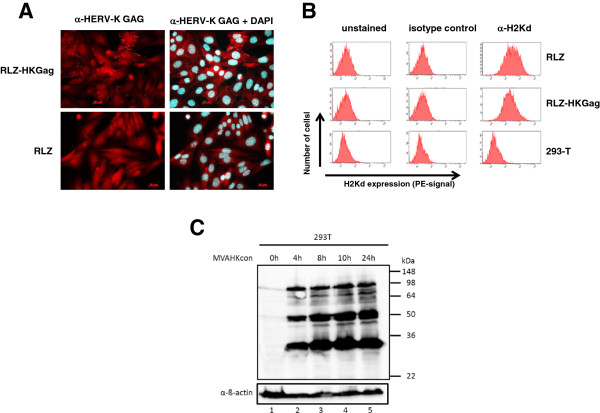
**Characterization of RLZ-HKGag cells and MVA-HK**_**con. **_**A**: Immun-fluorescence analysis of RLZ and RLZ-HKGag cells. Cells were fixed and stained either with an anti-HERV-K Gag antibody alone or in combination with DAPI. **B**: MHC class I expression MHC class I (H2Kd) expression was analyzed by flow cytometry either with an antibody directed against H2Kd or a control antibody of the same isotype. Human 293 T cells were used as negative control. **C**: Western Blot analysis of MVA-HK_con_-infected 293 T cells 293 T cells were infected at an MOI of 5 with MVA-HK_con_ and cell lysates were prepared at the indicated time points. HERV-K GAG was identified with the HERV-K GAG monoclonal antibody followed by chemiluminescent detection. Detection of ß-actin was used as loading control.

The development of HERV-K-specific vaccines is hampered by the availability of an appropriate animal model because HERV-K is exclusively expressed in humans. To bypass this limiting fact, we previously have generated a syngeneic mouse model for HERV-K Env [9] and expanded this model to HERV-K Gag. Murine renal carcinoma cells (Renca) were genetically altered to express E. coli beta-galactosidase (RLZ cells) [10] and the HERV-K Gag gene was introduced by retroviral transduction (RLZ-HKGag cells). The engineered cell line RLZ-HKGag is positive for Gag expression, as illustrated by a punctured positive staining shown by immunofluorescence analysis (Figure 1A upper left panel). To confirm that Gag expression is not silenced during cell propagation, we passaged the cells *in vitro* and *in vivo* and investigated cell lysates for Gag expression by Western blot analysis. The cells could be passaged without silencing of Gag expression *in vitro* and *in vivo* (data not shown). Both cell lines (RLZ and RLZ-HKGag) expressed MHC class I at similar levels (Figure 1B). Subcutaneous application of RLZ-HKGag cells into syngeneic BALB/c mice resulted in local tumors and intravenous application of cells gave rise to pulmonary metastases, which were detectable by X-gal staining upon excision of the lungs (data not shown).

As a vaccine vector, we chose the modified vaccinia virus Ankara (MVA). MVA is a highly attenuated and replication-deficient strain of vaccina virus that has been demonstrated to be safe for humans and is widely and increasingly considered as the vaccinia virus strain of choice for clinical investigation because of its excellent safety profile. Despite its inability to replicate in most mammalian cells, MVA still efficiently expresses viral and recombinant genes making it a potent antigen delivery platform. We inserted the coding sequence of HERV-K gag into the MVA genome via homologous recombination [11]. The recombinant MVA (MVA-HK_con_; named after the consensus gene) encodes the consensus HERV-K Gag-Pr-Pol gene [12] controlled by a strong early/late promoter (mH5). MVA gene expression is cascade like and the use of synthetic promoters that combine early and late elements is well established to maximize transgene expression [13]. Infection of 293 T cells at an MOI of 5 showed a typical early/late expression pattern of HERV-K Gag with high amounts of the precursor protein (90 kDa) and processed GAG proteins (50 and 30 kDa) (Figure 1C). In addition, we were able to show that infected cells produce virus-like HERV-K particles that bud from the cells [11].

### Vaccination with MVA-HK_con_ delays the tumor growth of subcutaneous tumors

In addition to be used as a tumor specific antigen, HERV-K Gag might be used as a novel HIV vaccine. In contrast to the rapidly mutating HIV-1 genome, HERVs are cellular genes that are not prone to mutation. HERV-K gene products are described to be overexpressed in HIV-infected individuals, and T-cell responses that are effective in lowering the HIV-1 viral load are potential therapeutic vaccine targets. So it is envisioned that a vaccine directed against HERV-K might also be valuable for the treatment of HIV-infected patients [14,15].

**Figure 2:**
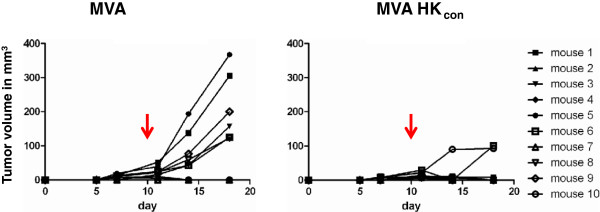
**Specificity of the vaccination in a subcutaneous tumor model.** Two groups of BALB/c mice (n = 10) were injected subcutaneously with 10^6^ RLZ-HKGag cells and immunized with either 10^8^ IU/mouse MVA-HK_con_ or 10^8^ IU/mouse MVA i.m. on day 10. Tumor size was monitored by caliper measurements. The arrow indicates the vaccination on day 10.

We tested the experimental vaccine in a therapeutic setting by starting with the subcutaneous injection of 1x10^6^ RLZ HKGag cells into the flanks of BALB/c mice on day 0. Ten days later, after the cells were able to form palpable tumors, the mice were vaccinated intramuscularly with either MVA-HK_con_ or MVA (10^8^ IU/mouse; n = 10). The initial tumor volume was similar in all mice and was monitored by caliper measurements. Out of ten mice, six mice in the MVA-vaccinated control group developed tumors. However, after MVA-HK_con_ vaccination, only two mice had papable tumors that were significantly smaller on day 18 after transplantation than those of MVA-vaccinated mice (Figure 2). Although this model showed a very high variation and did only show unconvincing statistically significant differences (p = 0.044), it nonetheless suggested that HERV-K GAG directed immune responses were generated and able to constrain tumor growth.

### Vaccination with MVA-HK_con_ reduces the number of lung metastases

**Figure 3:**
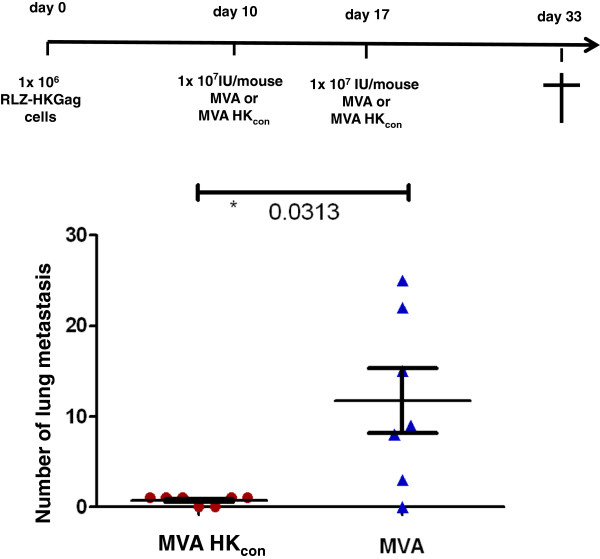
**Specificity of the vaccination in a lung metastasis tumor model.** Two groups of BALB/c mice (n = 7) were injected i.v. with 10^6^ RLZ-HKGag cells and immunized with 10^7^ IU/mouse MVA-HK_con_ or MVA i.m. on day 10 and 17. The animals were sacrificed on day 33, lungs were removed, and stained with X-Gal. The number of tumor nodules was counted on the surface of the lungs. Data for each mouse are shown. The p-value of 0.0313 shows significance.

We validated the above data in a second experiment using the systemic application of Renca cells which leads to the formation of lung metastasis instead of subcutaneous tumors. Therefore, 1 × 10^6^ RLZ-HKGag cells were applied intravenously into the tail vein of BALB/c mice. The application of cells was defined as day 0 of the experiment. Ten days later, after the cells have had the chance to colonize the lung, the mice were vaccinated intramuscularly with either MVA-HK_con_ or MVA (10^7^ IU/mouse; n = 7) (Figure 3). The vaccination was repeated on day 17 (10^7^ IU/mouse). On day 33, mice were sacrificed and the lungs were excised, fixed and stained for beta-galactosidase. In five out of seven MVA-HK_con_-vaccinated animals only one stainable metastasis could be found. Two animals were completely metastasis free (Figure 3). On the contrary MVA-vaccinated animals had a much higher tumor burden. Except for one mouse, all mice developed lung metastasis as quantified in Figure 3. Statistical analysis shows that there was a statistically significant difference in the number of metastases with a p-value of 0.031 (Figure 3). In summary, vaccination with MVA-HK_con_ severely decreased the outgrowth of pulmonary tumors.

## Discussion

Here, we assessed an experimental vaccine candidate for its efficacy to eliminate autologous HERV-K positive cells *in vivo*. We chose a vaccine candidate known to elicit strong T cell responses *in vivo*. The vaccine candidate is based on the modified vaccinia virus Ankara (MVA), a highly attenuated vaccinia virus strain with a high safety profile suitable for clinical application in immunosuppressed patients [16,17]. MVA does not replicate in human cells but shows high protein expression and, consequently, has a very good safety profile without compromising vaccination efficiency. As shown before, MVA vaccination generates antigen specific cellular immunity that is believed to be responsible to clear antigen expressing cells [9].

Vaccine efficacy testing of the recombinant MVA-HK_con_ was performed in a surrogate mouse model, using a syngeneic mouse tumor cell line which was genetically engineered to express HERV-K Gag. Since HERV-K expression is normally repressed in humans, it is expected that humans are not tolerant to HERV-K gene products and so our simplistic animal model might be appropriate as well. Vaccinations were performed in a therapeutic setting after the establishment of tumor nodules in the mice testing two tumor application routes. Subcutaneous tumor growth and the formation of pulmonary metastasis in the mice after i.v. application were significantly reduced. These data indicate a therapeutic effect of the HERV-K GAG-directed vaccination.

Two therapeutic applications can be envisioned for this vaccine candidate. First, as described before for a HERV-K Env expressing MVA, MVA-HK_con_ could be used as a tumor vaccine [9]. However, in addition HERV-K Gag might be used as a surrogate target to develop an HIV vaccine. Despite significant progress and several clinical trials, a safe and effective AIDS vaccine is still elusive. The virus evolved unique ways of evading the immune system, and the human body seems to be incapable of mounting an effective immune response against it. The retrovirus HIV is hypervariable due to its high mutation rate and the ability to recombine; for vaccine development this means aiming at a moving target. Recently, several reports indicate that HERV-K is activated by HIV infection (see review by van der Kuyl [18] and cell-mediated immune responses directed against HERV-K are effective in lowering HIV-1 viral loads and correlated with control of HIV-1 viremia [14,15,19]. In addition, antibodies directed against HERV-K were detected in 70 to 80% of patients with HIV viremia. In contrast, only 2% of normal healthy controls were tested anti-HERV-K positive [20]. Then again the absence of HERV-specific antibodies in AIDS patients has been reported as well [21-23].

A direct link between HERV-K activation and HIV infection was suggested recently [24]. Gonzalez-Hernandez and colleagues reported that addition of recombinant HIV-1 Tat protein to Jurkat cells caused a 13-fold increase in HERV-K gag RNA transcripts and a 10-fold increase in treated primary lymphocytes [24]. However, the correlation between HIV infection and HERV-K expression is still very controversial. Clear data showing HERV-K expression in HIV-infected cells from patients are not yet available and expression of HERV-K in CD8^+^ cells cannot be excluded [25]. In addition, HERV-K *pol* expression has been described after herpes virus, hepatitis B and C virus infections [18], indicating a more general mechanism of activation. Quantification of HERV-K RNA in HIV-infected patients under HAART showed a correlation of HERV-K and HIV viral load, however an effect of HAART on HERV-K cannot be excluded [26]. In general, no significant correlation has been described between HIV viral load, CD4^+^ T cell counts and HERV-K protein titers [18].

In contrast, Garrison *et al.*[19] demonstrated the stimulation of HERV-specific T cell responses in HIV-positive participants by ELISPOT detection [19]. Interestingly, HERV-specific T-cell responses inversely correlated with HIV-1 plasma viral load [19]. Another study showed that HIV infected individuals (Long-term nonprogressors (LTNPs) or elite controllers), who control HIV-1 viremia without highly active antiretroviral therapy (HAART) had stronger and broader HERV-specific T cell responses than HAART-suppressed patients, virologic noncontrollers, immunologic progressors, and uninfected controls. In addition, the magnitude of the anti-HERV T cell response inversely correlated with HIV-1 viral load and associated with higher CD4 T cell counts in untreated patients. This suggests a beneficial effect of anti-HERV immunity in the control of chronic HIV-1 infection [14]. Moreover, a HERV-K–specific CD8+ T cell clone has been described to be able to eliminate cells infected with a panel of globally diverse HIV-1, HIV-2, and SIV isolates in vitro, indicating that HERV-K–specific T cell responses might be involved in the control of HIV-1 infections [15].

Although still discussed controversially, a lot of data indicate that HIV-1 infection leads to the expression of otherwise repressed HERV-K and consequently to the stimulation of HERV-specific immune responses which in turns might help to control the HIV infection. However, still basic issues need to be clarified. Are HERV-K epitopes expressed significantly and exclusively in HIV-infected cells; T cells as well as monocytes. Another open issue is the next step towards clinical development. Side effects of an HERV-K-specific vaccination can only be studied in humans.

Recent observations of HERV-K expression in embryonic and induced pluripotent stem cells [27] might also be of concern, however embryonic stem cells are located in an immune protected tissue and should not be recognized by HERV-K-specific immune responses. However, potential hazardous HERK-K expression in hematopoietic stem cells still needs to be analyzed. Primates encode very similar endogenous retroviral genomes (SERV) and could be used as a model for safety studies [28-31]. Vaccination of rhesus macaques which carry SERV-K with SERV-K Gag or Env induced T cell responses without vaccine-related pathogenicity [31].

HERV-K Env expression has also been observed in diverse types of human tumors. We recently showed proof of principle that vaccination directed against the HERV-K envelope protein had anti-tumor activity [9]. A HERV-K Gag directed vaccine might therefore be used as a tumor vaccine and might have in addition an application as a HIV vaccine.

## Methods

### Cell culture and virus

HEK 293 T (ATCC: CRL-1573) and Renca (CRL-2947) cells were cultured in complete Dulbecco’s modified Eagle’s medium (DMEM) containing 10% fetal bovine serum, penicillin (50 U/ml), streptomycin (50 μg/ml), Zeocin (RLZ cells; 50 μg/ml) and L-glutamine (2 mM), and propagated by standard techniques. For the generation of the RLZ-HKGag cell line, MLV-based retroviral vectors were produced encoding a codon-optimized HERV-K GAG gene via the pBabe-puro vector. For selection of transduced cells, 1 μg/ml puromycin was added to the RLZ-HKGag medium.

The recombinant virus MVA-HERV-K_con_ was generated as described before [11].

### Western blot

Western blot was performed with a BIO-Rad semi-dry blotter. Proteins separated by SDS-PAGE were blotted onto PVDF membranes with 50 mM sodium borate pH 9.0, 20% methanol, and 0.1% SDS at 100 mA per membrane for 75 min. Afterwards, membranes were blocked with Roti-Block™ and proteins were detected with α-HERV-K capsid monoclonal antibodies [32] and the ECL detection system (Amersham, Freiburg).

### Immunofluorescence staining of cells

Cells were fixed with 2% paraformaldehyde and HERV-K Gag was detected after successive incubations with α-HERV-K capsid monoclonal antibodies [32] and a FITC-coupled goat anti-mouse IgG antibody (DAKO, Hamburg, Germany). Additionally cells were stained with DAPI. Cells were examined with a 400× magnification.

### Flow cytometry

Cells were incubated with the indicated antibodies for 60 min at 4°C at the appropriate dilution as determined by previous titration. Cells were washed with FACS buffer and analyzed by flow cytometry. At least 10.000 events were acquired with an LSRII instrument (BD Biosciences) and analyzed using FACS Diva Software. Matching isotype control antibodies were used as negative controls.

### Animal experiments

Female specific pathogen-free 6–8-week-old BALB/c mice were purchased from Harlan. For tumor transplantations, RLZ-HKGag cells were grown to 60–80% confluence and detached from the culture dish via trypsin digestion. Cells were washed three times with PBS and resuspended in PBS at a density of 1 × 10^6^ cells/100 μl for vaccination experiments. Indicated cell numbers were injected subcutaneously into the flanks or i.v. into the tail vein of mice using standard techniques. Mice were immunized with the indicated amounts of recombinant MVA intra-muscularly into the quadriceps muscles of the hind limbs and then weighed every 3 days. Mice were sacrificed at the indicated time points, and the lungs were prepared and incubated in fixing solution (0.2% glutaraldehyde, 2% formaldehyde in PBS) overnight at 4°C. For X-Gal staining, lungs were incubated in staining solution (5 mM potassium ferrocyanide, 5 mM potassium ferricyanid, 1 mM MgCl_2_, 1 mg/ml X-Gal in PBS) for 24 h. Stained lungs were stored in PBS containing 4% formaldehyde and metastases were counted. All experiments were performed in accordance to legal requirements (Regional Council Darmstadt).

## Competing interests

The authors declare that they have no competing interests.

## Authors’ contributions

BK and KF carried out the studies and helped draft the manuscript. KS participated in the design of the study and helped draft the manuscript. BS conceived the study, and participated in its design and coordination and drafted the manuscript. All authors read and approved the final manuscript.
